# Genome-wide association study of maize resistance to *Pythium aristosporum* stalk rot

**DOI:** 10.3389/fpls.2023.1239635

**Published:** 2023-08-17

**Authors:** Mengwei Hou, Yanyong Cao, Xingrui Zhang, Shulin Zhang, Tengjiao Jia, Jiwei Yang, Shengbo Han, Lifeng Wang, Jingjing Li, Hao Wang, Lili Zhang, Xiaolin Wu, Canxing Duan, Huiyong Li

**Affiliations:** ^1^ Institute of Cereal Crops, Henan Academy of Agricultural Sciences, Zhengzhou, China; ^2^ Institute of Crop Sciences, Chinese Academy of Agricultural Sciences, Beijing, China; ^3^ College of Biology and Food Engineering, Anyang Institute of Technology, Anyang, China; ^4^ College of Life Science, Henan Agricultural University, Zhengzhou, China

**Keywords:** maize stalk rot, *Pythium aristosporum*, genome-wide association study, resistance gene, leucine-rich repeat receptor-like kinase, virus-induced gene silencing

## Abstract

Stalk rot, a severe and widespread soil-borne disease in maize, globally reduces yield and quality. Recent documentation reveals that *Pythium aristosporum* has emerged as one of the dominant causal agents of maize stalk rot. However, a previous study of maize stalk rot disease resistance mechanisms and breeding had mainly focused on other pathogens, neglecting *P. aristosporum*. To mitigate crop loss, resistance breeding is the most economical and effective strategy against this disease. This study involved characterizing resistance in 295 inbred lines using the drilling inoculation method and genotyping them via sequencing. By combining with population structure, disease resistance phenotype, and genome-wide association study (GWAS), we identified 39 significant single-nucleotide polymorphisms (SNPs) associated with *P. aristosporum* stalk rot resistance by utilizing six statistical methods. Bioinformatics analysis of these SNPs revealed 69 potential resistance genes, among which *Zm00001d051313* was finally evaluated for its roles in host defense response to *P. aristosporum* infection. Through virus-induced gene silencing (VIGS) verification and physiological index determination, we found that transient silencing of *Zm00001d051313* promoted *P. aristosporum* infection, indicating a positive regulatory role of this gene in maize’s antifungal defense mechanism. Therefore, these findings will help advance our current understanding of the underlying mechanisms of maize defense to Pythium stalk rot.

## Introduction

Maize (*Zea mays* L.) is a vital crop in China with the highest yields, surpassing wheat and rice in acreage. However, owing to the limited arable land area, continuous cultivation of the same maize variety leads to the accumulation of pathogenic microorganisms in the soil. This results in numerous soil-borne diseases that seriously affect the quality and yield, with stalk rot being a particularly devastating and prevalent disease widespread in maize production (Jackson et al., 2009; Yang et al., 2010; Duan et al., 2022). Although stalk rot disease severities and accompanying yield losses vary across different countries and regions, recent changes in climate and cultivation methods have made it a major threat to maize production (Duan et al., 2019). The primary fungal culprits responsible for maize stalk rot are *Fusarium* spp. and *Pythium* spp. (Song et al., 2015), among which *Pythium aristosporum* has recently emerged as a highly aggressive pathogen (Liu et al., 2019; Ding et al., 2021). Conventional control strategies using fungicides or biological agents have shown limited success against the soil-borne pathogens that cause stalk rot. Therefore, the most effective and sustainable disease control approach is selective breeding of Pythium stalk rot-resistant maize varieties (Duan et al., 2019).

The resistance to stalk rot in maize was considered to be a quantitative trait and conferred by multiple genes or quantitative trait loci (QTLs) (Pè et al., 1993; Toman and White, 1993; Yang et al., 2010; Wang et al., 2017; Ye et al., 2019). To date, only a few of Pythium stalk rot resistance genes have been mapped and designated, including *Rpi1* (Yang et al., 2005), *RpiQI319-1* and *RpiQI319-2* (Song et al., 2015), and *RpiX178-1* and *RpiX178-2* (Duan et al., 2019). However, these genes are specific to maize resistance to *P. inflatum*, and only limited information was available to maize breeders regarding genetic and molecular analysis of resistance to maize stalk rot caused by other *Pythium* spp., especially *P. aristosporum*.

Over the last decade, next-generation sequencing (NGS) technologies have revolutionized crop breeding. NGS enables rapid acquisition of a vast number of SNPs throughout the genome, offering greater DNA variation compared to traditional markers. Now, NGS has been widely used for linkage mapping, genome-wide association study (GWAS), marker-assisted selection (MAS), genomic selection (GS), domestication, and population structure analysis in crops (Zhou et al., 2015; Bhat et al., 2016; Varshney et al., 2016; Scheben et al., 2017; Wang et al., 2018; Jamil et al., 2019). Using these technologies has facilitated the mapping and cloning of numerous important genes/QTLs (Huang et al., 2010; Sun et al., 2017; Lu et al., 2018), providing abundant operational targets for molecular design breeding and gene modification (Peleman and van der Voort, 2003).

GWAS has recently gained significant attention due to its ability to analyze the genetic basis of complex traits using existing natural populations, making it more time-efficient than traditional linkage analysis. This approach has been successfully applied to crops like rice, maize, and soybean (Zhao et al., 2011; Yang et al., 2014; Cao et al., 2017). GWAS enables simultaneous association analysis of multi-trait phenotype data across multiple environments and time points, facilitating the detection of millions of SNPs at once. However, GWAS results can be easily influenced by population structure and rare variants in natural populations. To enhance the power of identifying phenotype–genotype associations, various analytical models have been developed, including the Bayesian-information and Linkage-disequilibrium Iteratively Nested Keyway (Blink; Huang et al., 2019), Fixed and random model Circulating Probability Unification (FarmCPU; Liu et al., 2016), General Linear Model (GLM; Friston et al., 1994), Mixed Linear Model (MLM; Yu and Buckler, 2006), Multiple Loci Mixed Model (MLMM; Segura et al., 2012), and Settlement of MLMs Under Progressively Exclusive Relationship (SUPER; Wang et al., 2014).

In this study, we investigated 295 maize inbred lines in two environments for disease severity index (DSI) of stalk rot symptom phenotypes. Genotyping-by-sequencing (GBS) technology was used to genotype these inbred lines. Subsequently, we conducted GWAS analysis to identify SNPs significantly associated with resistance to *P. aristosporum* stalk rot (PASR). The detected significant loci were subjected to bioinformatic analysis to identify candidate genes for disease resistance. Finally, the expression of the candidate genes was verified using quantitative real-time fluorescence PCR (qRT-PCR), and their functions were verified through virus-induced gene silencing (VIGS). Therefore, our study can deepen the understanding of the genetic mechanisms underlying stalk rot resistance in maize. Additionally, it provides valuable research methods and candidate genes for future molecular breeding strategies aimed at improving maize disease resistance.

## Materials and methods

### Plant materials and trail designs

A total of 295 maize inbred lines were assembled into a panel. The panel included 134 China core germplasms and 161 expired US plant variety protection inbred lines (provided by the China National Modern Corn Industry Technology System). The test materials of the associated panel were planted in Xinxiang (XX, 35.108°N,113.792°E) and Dancheng (DC, 33.646°N, 115.257°E) experimental stations of Henan Academy of Agricultural Sciences in 2021. The field experiment was arranged in a randomized complete block design with two replicates. Each inbred line was grown in two rows with 15 plants in each row, 0.60 m in row spacing, and 0.25 m in plant spacing. Resistant line Qi319 and susceptible line Y478 served as phenotyping controls, respectively.

### Fungal inoculation and disease symptoms evaluation

*Pythium aristosporum* strain T2 was preserved and propagated in our laboratory and cultured on fresh potato dextrose agar (PDA) plates (ca. 20 ml per plate) at 25°C in the darkness for 5–7 days. The inoculum was achieved by homogenization of five plates of flourish hyphal mats (approximately 125 ml) with kitchen blender, adjusting to a final volume of 200 ml with double-deionized sterile water (ddH_2_O).

#### Artificial inoculation of maize seedling roots

Three to four leaf-stage maize seedlings were transferred into 250 ml of 1/2 MS media (Sigma, Saint Louis, MO, USA) poured in a 24 × 24-cm culture dish (BioSharp Life Sci, Beijing, China). Maize roots were placed on the surface of the medium before the infection. Then, the root tips were inoculated with 20 μl of the freshly prepared inoculum, and mock-inoculated maize roots treated with PDA plug served as the control. The root systems were covered with a sheet of germination paper wetted with liquid 1/2 MS media and a piece of aluminum foil to maintain moisture and avoid light exposure and contamination from other potential organisms. Finally, the culture dish was covered with a zip lock bag (BioSharp) and transferred into the growth cabinet for another 48 h at 25/20°C (day/night).

Roots of the inoculated seedlings were scored at the disease regions at 24 hours post inoculation (hpi) or 48 hpi with a rating grade of 1–5 as previously described (Ye et al., 2019).

#### Artificial infection of maize stalks

Maize plants at the flowering stage or fifth-leaf stage (for target gene-silenced seedlings) were used for stalk inoculation based on a previously described protocol (Zhang et al., 2016; Cao et al., 2021) with modification. Maize plants were inoculated by punching a hole in the stem at the second or third internode above the soil line, followed by injection of 1 ml (50 μl for seedlings) of freshly prepared *P. aristosporum* inoculum. The wounds were sealed with Vaseline after inoculation. Then, the fields or the planting pots were fully irrigated to mimic growth condition favoring fungal growth and stalk rot disease development.

**Figure 1:**
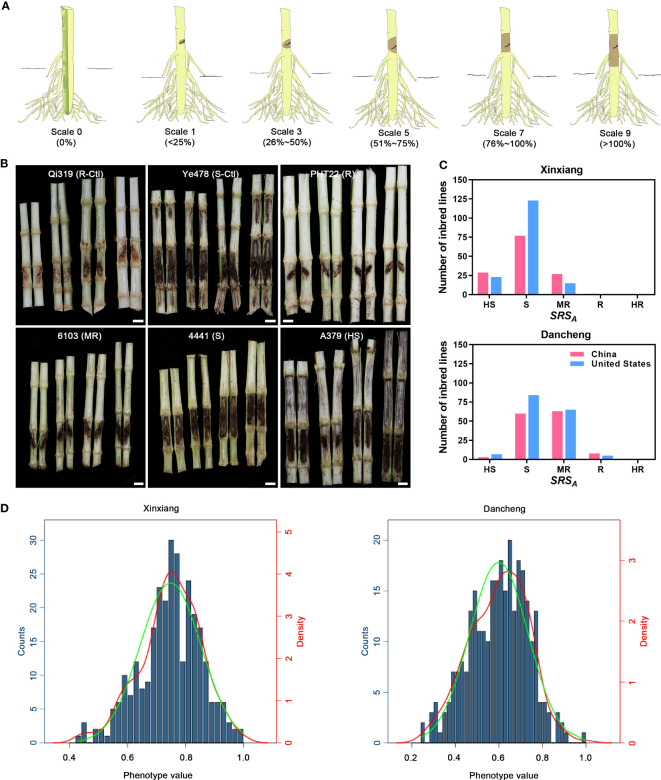
Phenotypic descriptions and evaluation of maize Pythium stalk rot resistance. **(A)** Schematic diagram of the classification of different scales of stalk rot symptoms. **(B)** The phenotypic characteristics of resistant (R), moderately resistant (MR), susceptible (S), and highly susceptible (HS) inbred lines. Qi319 and Ye478 served as resistant and susceptible control. **(C)** Histogram statistics of the number of inbred lines with different resistance levels in two environments, Xinxiang and Dancheng. **(D)** Histogram of phenotypic distribution of stalk rot in Xinxiang and Dancheng. The green curve indicates the standard normal curve and the red one indicates the density curve fitted according to the phenotypic distribution.

For the evaluation of stalk rot symptoms, 48 h (seedlings) or 40 days (adult plant) after inoculation, the inoculated internodes of the individual maize plants were split and symptoms were observed with scores of 0, 1, 3, 5, 7, and 9 according to a previously described classification standard (Duan et al., 2022) with adjustment. Scale 0, no detectable spread of the pathogen from inoculation site; scale 1, 1%–25% of inoculated internode is symptomatic; scale 3, 26%–50% of inoculated internode is symptomatic; scale 5, 51%–75% of inoculated internode is symptomatic; scale 7, 76%–100% of inoculated internode is symptomatic; scale 9, 100% of inoculated internode is symptomatic and the necrotic lesion spread to adjacent internodes (**Figure 1A**).

Two parameters were used to evaluate the symptoms of roots or stalks: the first one was SRS_A_ (stalk rot score on average) = $\;\sum_{1}^{n}SRS$ /n; the second one was [Disease severity index (%)] (DSI) = ∑ (grade × number of plants in grade) × 100/(maximum grade × total number of plants).

### Statistical analysis of phenotypic data

Normal distribution analysis and correlation analysis of phenotypic data were calculated by SPSS 20 statistical software (IBM Corp., Armonk, NY). QTL IciMapping software (Institute of Crop Science, Chinese Academy of Agricultural Sciences, Beijing, China) was used for variance analysis, best linear unbiased prediction of combined phenotype data, and the evaluation of broad-sense heritability (*H^2^*) (Li et al., 2008).

### Genotyping, population structure, kinship, and GWAS

Leaves of the seedling were collected and flash frozen in liquid nitrogen. A modified CTAB procedure was followed for DNA extraction in this study (Healey et al., 2014), and the DNA quality and concentration were verified by 1% agarose gel-electrophoresis and spectrophotometer before sequencing. The high-quality DNA samples were used to construct sequencing libraries as described in the previous study (Jia et al., 2019). All inbred lines were genotyped using GBS. Sequencing was completed using Illumina HiSeq 4000 (Illumina Inc., San Diego, CA, United States). After data filtering, the obtained high-quality sequencing data were aligned to the B73 reference genome (RefGen_V4). The SNPs were identified using SAMtools (Li et al., 2009). Markers with minor allele frequencies (MAF) less than 5%, missing rates greater than 20%, and heterozygous rates more than 10% were removed. Finally, the ANNOVAR software (https://www.openbioinformatics.org/annovar/) (Wang et al., 2010) was employed to annotate the identified SNPs.

The principal component analysis (PCA) was used to evaluate the genetic structure using the software genome-wide complex trait analysis (GCTA) (Yang et al., 2011).

GWAS is an effective approach for analyzing the genetic basis of complex traits. In this study, we used the Blink, FarmCPU, GLM, MLM, MLMM, and SUPER models implemented in GAPIT3 R package (Wang and Zhang, 2021). In addition, *p<* 10^−5^ was used to declare significant associations based on a previous study with optimization (Li et al., 2019), and FDR<0.05 was used to identify significant associations. Potential candidate SNPs were selected by the significance of the association. In maize inbred lines, LD decay may be slower and linkage blocks may extend more than 100 kb based on a previous study, in which GWAS-identified genes were identified based on the B73 genome sequence (Ching et al., 2002). The 100-kb regions flanking the left and right sides of each significant SNP were defined as QTL in this study. The genes within this window size were identified in MaizeGDB and UniProt according to the positions of the closest flanking significant SNPs or supporting intervals.

### Physiological and biochemical characteristics measurement

To assay the physiological and biochemical characteristics related to plant defense reaction post fungal infection, the roots and leaves of the seedlings were harvested at different time points post inoculation. The physiological and biochemical characteristics, including peroxidase (POD) activity (U/g), catalase (CAT) activity (U/g), and superoxide dismutase (SOD) activity (U/g), were measured with the assay kits of Comin Biotechnology Co., Ltd (Suzhou, China) following the manufacturer’s protocols.

H_2_O_2_ production in plants was spectrophotometrically measured using xylenol orange assay (Bindschedler et al., 2001) with the modified protocol described by Hwang and Hwang (2010)

### Quantitative real-time fluorescence PCR

Total RNA was isolated from different samples using TRIzol reagent (Invitrogen, Carlsbad CA, United States) and then treated with RNase-free DNase I (TaKaRa, Dalian, China). The first-strand cDNA was synthesized using 2.0 μg of total RNA per 20 μl reaction and an oligo (dT) primer. Tenfold diluted cDNA, a set of gene-specific primers (**Supplementary Table S1**), and TB Green^®^ Premix Ex Taq^™^ II mix (TaKaRa, Dalian, China) were mixed for qRT-PCR to determine the transcriptional levels of the maize genes on the CFX96^™^ real-time PCR detection system (Bio-Rad, Hercules, CA, United States). The expression level of GAPDH mRNA was determined and used as an internal control. The relative expression level of each gene was calculated using the 2^–ΔΔCt^ method (Livak and Schmittgen, 2001). Differences between the treatments were then analyzed using Student’s *t*-tests. All experiments were carried out at least three times.

### Virus-induced gene silencing

An improved brome mosaic virus (BMV)-derived VIGS vector (Ding et al., 2018) was used to knock down the target genes. A DNA fragment of 253 bp, representing the *Zm00001d051313* gene, was amplified using specific primer pairs (**Supplementary Table S1**) to construct the VIGS vector. To evaluate the genetic stability of foreign inserts in the VIGS vector during RNA silencing in plants, the folding structure formed by the full-length BMV RNA3 sequence in pF3-13m of pBMV-CP5 vector or with gene fragment inserts were predicted using a deep learning-based RNA secondary structure prediction tool MXFold2 (Sato et al., 2021; http://www.dna.bio.keio.ac.jp/mxfold2/). Only the vector harboring foreign gene fragment inserts without changing the stability of BMV RNA3 was chosen for subsequent analysis (**Supplementary Figure S1**). The resulting constructs BMV-d051313 and the control BMV-GFP were then transformed into the *Agrobacterium tumefaciens* strain C58C1. The *Nicotina benthamiana* leaves were infiltrated with *A. tumefaciens* cultures and collected for BMV virion preparation, as described previously (Zhu et al., 2014).

The third leaves of the three-leaf-stage Va35 plants were rub-inoculated with approximately 20 μg of partially purified BMV virion. More than 20 seedlings were used for each treatment, and the inoculated plants were grown inside a growth chamber at 18/20°C (day/night) for 7–10 days before being challenged with *P. aristosporum*. Systemically infected maize leaves (or equivalent maize leaves of mock-inoculated plants) from BMV-d051313 or BMV-GFP inoculated plants were harvested from individual plants at 7 and 10 days post inoculation (dpi) and subjected to qRT-PCR to evaluate the efficiency of gene silencing.

## Results

### Phenotype descriptions and evaluation of the resistance to maize stalk rot

To rapidly screen and evaluate the resistance of maize plants to stalk rot, we planted 295 maize inbred lines collected in this study in two environments for artificial inoculation. The inoculated plant stalks were then scored for symptom development in the diseased region using rating scales of 0, 1, 3, 5, 7, and 9 (**Figure 1A**). The data indicated that none of the tested lines were fully immune to *P. aristosporum*. **Figure 1B** shows the phenotypic characteristics of inbred lines PHT22, 6103, 4441, and A379, representing resistant, moderately resistant, susceptible, and highly susceptible lines, respectively. Additionally, symptomatic characteristics of Qi319 and Ye478, which served as resistant and susceptible control lines, were also displayed. Although there were variations in the phenotypic identification of resistance to *P. aristosporum* among the 295 inbred lines in the two experimental environments, the overall trend remained consistent. Furthermore, we observed variations in performance between the inbred lines from China and United States (**Figure 1C**).

In Xinxiang and Dancheng, a total of 42 (14.24%) and 141 (47.8%) accessions, respectively, showed moderate resistance, resistance, or high resistance to stalk rot. In Xinxiang, 253 (85.76%) inbred lines were susceptible and highly susceptible, and 52 (17.63%) were highly susceptible. However, in Dancheng, these percentages were 154 (52.2%) and 9 (3.05%), respectively (**Supplementary Table S2**). Based on these results, we speculated that the growing environment in Xinxiang is more conducive to the survival and spread of stalk rot than that in Dancheng.

To determine the resistance level in 295 maize inbred lines, we combined DSI values with disease resistance scores and plotted histograms of phenotypic traits by calculating phenotypic disease resistance data from both locations. The skewness and kurtosis of the phenotypic distribution plots for both environments indicated a normal distribution for the phenotypic data, and the combination environment DSI (%) ranged from 16.67% to 100%, suggesting the abundant phenotypic variation in this panel. In a single environment, the broad-sense heritability of Xinxiang was the highest (0.86), followed by Dancheng (0.84), and the combination of both had the lowest (0.68). Regarding phenotype traits, the heritability of a combination environment was lower than that of a single environment. The experiment revealed significant correlations (*p<* 0.01) among the environment (E), genotype variation (G), and G*E (genotype-by-environment interaction), suggesting that resistance traits to stalk rot are controlled by multiple genes and can be localized for further analysis by GWAS (**Figure 1D**; **Supplementary Table S3**).

### Genotype by sequencing and analysis of population structure

In this study, we obtained a total of 201.18 Gb of data from 295 maize inbred lines using GBS, with an average of 0.68197 Gb per sample. The sequencing quality was high, with Q20 ≥ 91.16% and Q30 ≥ 86.22%. The population showed an average alignment rate of 98.88%, and an average genome sequencing depth of 12.07×. From the sequencing data, we identified a total of 4,138,215 SNPs in the 295 inbred maize lines. Finally, we obtained a total of 217,933 SNPs for subsequent GWAS analysis after filtering SNPs with MAF > 0.05 and a missing rate< 0.20, as reported in previous studies (Jia et al., 2020). Among these SNPs, most (160,874) were located in the intergenic region, 18,553 in the intronic and non-coding regions, whereas 3,221 synonymous SNPs, 3,801 nonsynonymous SNPs, 150 stop-gain, and 21 stop-loss mutations were found in the exonic regions (**Supplementary Table S4**).

We performed PCA based on the genotype data of 295 inbred lines. The results of PCA showed that the population could be divided into three subgroups, similar to the results of a previous study (Jia et al., 2020) (**Supplementary Figure S2**). Although the same analysis tool was used, the population structure in this study differed from two other reports, where more representative germplasms were collected for population composition analysis (Wang et al., 2020; Li et al., 2022).

### Significant loci in association mapping

**Figure 2:**
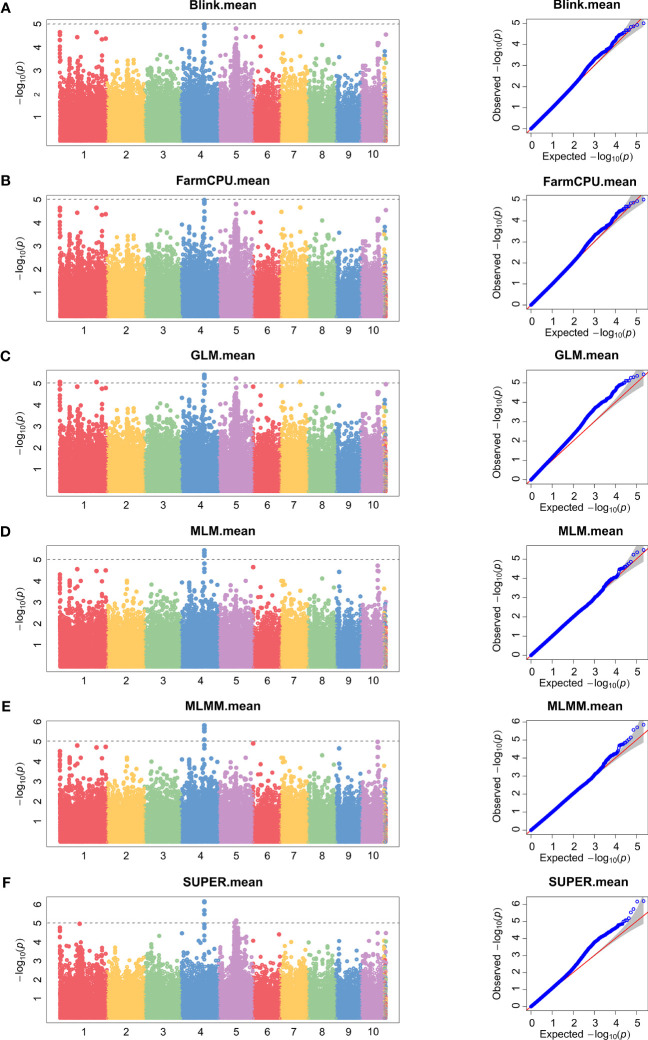
Manhattan and Quantile–Quantile plots of SNPs significantly associated with stalk rot resistance using different models in Xinxiang. **(A)** Bayesian-information and Linkage-disequilibrium Iteratively Nested Keyway (Blink). **(B)** Fixed and random model Circulating Probability Unification (FarmCPU). **(C)** General Linear Model (GLM). **(D)** Mixed Linear Model (MLM). **(E)** Multiple Locus Mixed linear Model (MLMM). **(F)** Settlement of MLM Under Progressively Exclusive Relationship (SUPER). Different colors in the Manhattan plots represent different chromosomes in maize.

**Figure 3:**
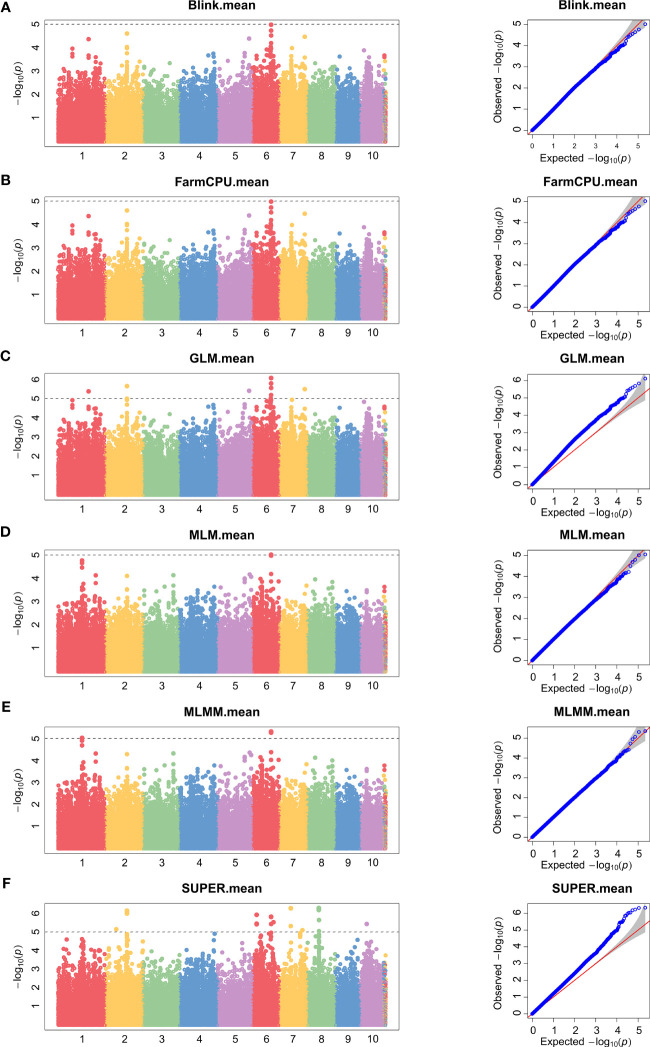
Manhattan and Quantile–Quantile plots of SNPs significantly associated with stalk rot resistance using different models in Dancheng. **(A)** Blink. **(B)** FarmCPU. **(C)** GLM. **(D)** MLM. **(E)** MLMM. **(F)** SUPER. Different colors in the Manhattan plots represent different chromosomes in maize.

To minimize population structure-related false positives, we employed six models (Blink, FarmCPU, GLM, MLM, MLMM, and SUPER models) for GWAS analysis. **Figures 2** and **3** show the Manhattan chart and Quantile–Quantile plot (Q–Q plot) of GWAS analysis at two locations, Xinxiang and Dancheng. After several tests, setting -log10 (*P*) > 5 as the screening threshold for the best significantly associated SNPs, we identified a total of 39 significantly associated SNPs in two environments using the six models (**Supplementary Table S5**). Among them, 15 SNPs were significantly associated with stalk rot at the experimental site in Xinxiang, with the highest number of significant SNPs (six SNPs) found on chromosome 4. In contrast, we detected 24 significant SNPs in Dancheng, with the highest number (9 SNPs) on chromosome 6.

**Figure 4:**
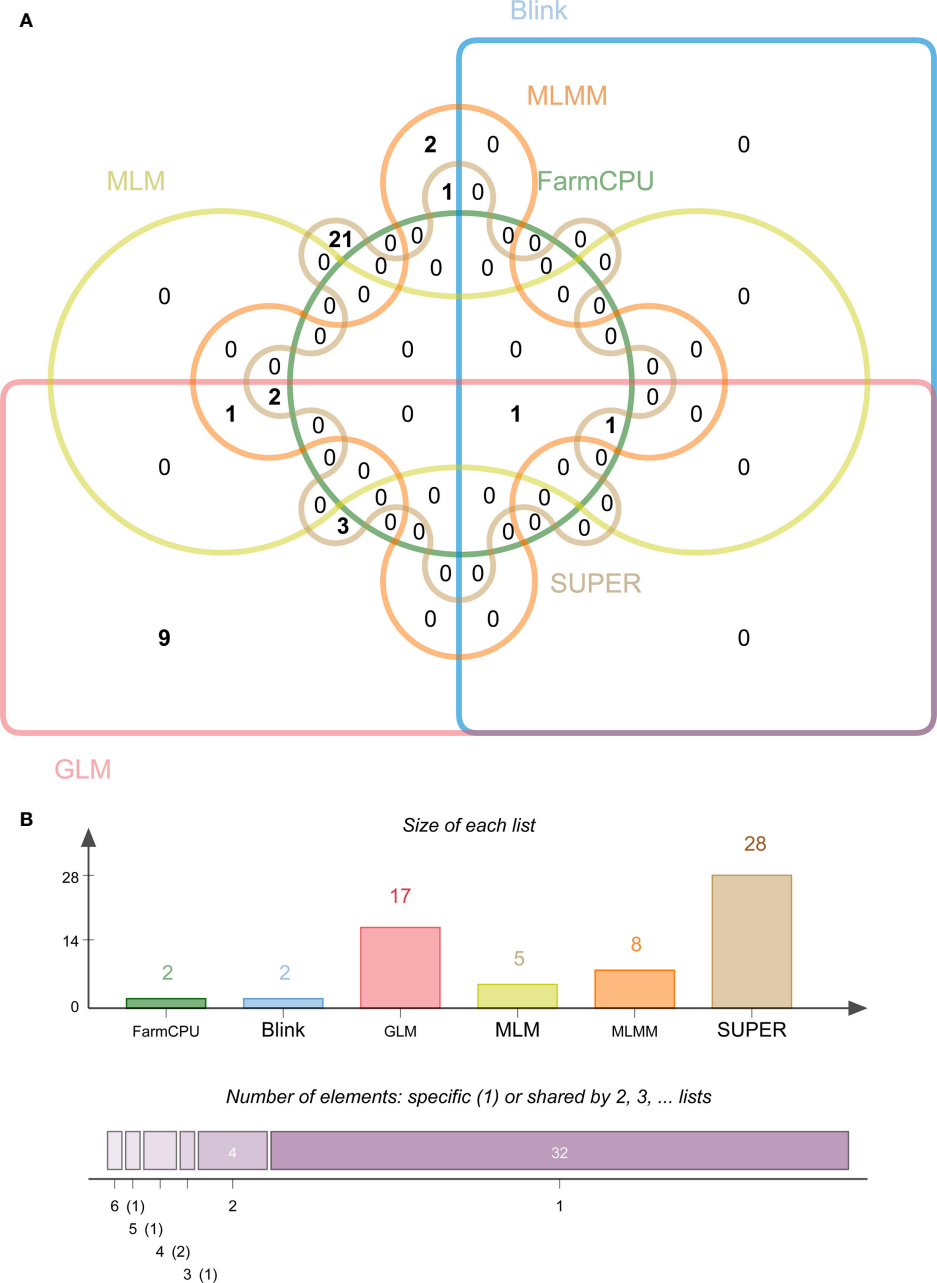
Statistical analysis of maize stalk rot resistance significantly associated SNPs identified by different models. **(A)** Venn plots of significant SNPs for Pythium stalk rot resistance identified by six methods. **(B)** Statistics of the number of significant SNPs using six different models (*P* > 10^-5^).

Among the 39 significantly associated SNPs, the SUPER model identified the most significant SNPs (28), followed by the GLM model (17), then MLMM, MLM, Blink, and FarmCPU with eight, five, two, and two SNPs, respectively (**Figure 4**). Interestingly, we found that only one SNP (S4_153228905) was jointly detected by Blink (*p* = 9.67E-06), FarmCPU (*p* = 9.67E-06), GLM (*p* = 3.61E-06), MLM (*p* = 3.36E-06), MLMM (*p* = 1.42E-06), and SUPER (*p* = 1.60E-06) models. Additionally, we detected another SNP (S6_121809944) under the remaining five models, except the SUPER.

### Candidate genes analysis

Subsequently, we screened candidate genes within a 200-kb region encompassing the significantly associated SNPs (100 kb upstream and 100 kb downstream). We performed functional annotation of the candidate genes using the UniProt database (**Supplementary Table S6**). The results showed that 69 genes were associated with 39 significantly associated SNPs. Besides the uncharacterized protein, the GWAS predicted genes encoding LRR-RLKs (leucine-rich repeat receptor-like kinase), L-type LecRLKs (lectin receptor-like kinase), PR (pathogenesis-related) proteins, hormone-responsive proteins, signal transduction elements, structural components of plant cell walls (membranes), transcriptional factors, ubiquitin ligase family proteins, secondary metabolites associated with plant defense regulators, and others (**Supplementary Table S6**). Among these, three genes (*Zm00001d051313*, *Zm00001d051314*, and *Zm00001d051315*) associated with S4_153228905 were jointly detected by six models and the MAF value is 0.095 (**Supplementary Table S5**). Additionally, the genes *Zm00001d037332* and *Zm00001d037333* were jointly detected by the five models and were related to S6_121809944. Because of the lack of functional annotation of *Zm00001d051315* in the database, it was excluded from the subsequent qRT-PCR experiments. To verify their expression levels, we performed qRT-PCR for the remaining four candidate genes in Ye478 and Qi319 seedlings. The results showed similar expression patterns of the four candidate genes in the resistant inbred line Qi319, exhibiting significantly higher expression abundance at different time points compared to the mock group. Conversely, in the susceptible line Ye478, we observed no significant differences or even opposite trends for *Zm00001d037332* and *Zm00001d037333* in the expression profiles (**Supplementary Figure S3**).

Based on the above-mentioned results, we finally selected S4_153228905-related candidate genes identified by all six models simultaneously for subsequent validation experiments. The candidate genes *Zm00001d051313* and *Zm00001d051314* were annotated as “leucine-rich repeat (LRR) family protein” and “putative receptor-like protein kinase” in the functional database, respectively. Both genes were potentially associated with plant response to pathogenic microbe infestation. The LRR family encodes LRR-RLKs that were directly related to disease resistance in various crops (Dievart et al., 2020; Si et al., 2021; Chen et al., 2023). The LRR-RLKs are key membrane receptor proteins in plants, which play critical roles in signal recognition, intercellular communication, and cellular responses to environment stress. Moreover, subcellular localization prediction for *Zm00001d051313* indicated its presence in the cell membrane. Finally, we predicted the signal peptide using the SignalP-5.0 Server (https://services.healthtech.dtu.dk/service.php?SignalP-5.0) and found that the Signal peptide (Sec/SPI) value of this protein is 0.9847. This indicates that the signal peptide is very likely to be present with a cleavage site at position 26–27 and a probability of 80.74% (**Supplementary Figure S4**). Therefore, we determined *Zm00001d051313* as the final candidate gene for further investigation of its function.

### Transient silencing of the LRR-RLK gene facilitates *Pythium aristosporum* infection in maize plants

**Figure 5:**
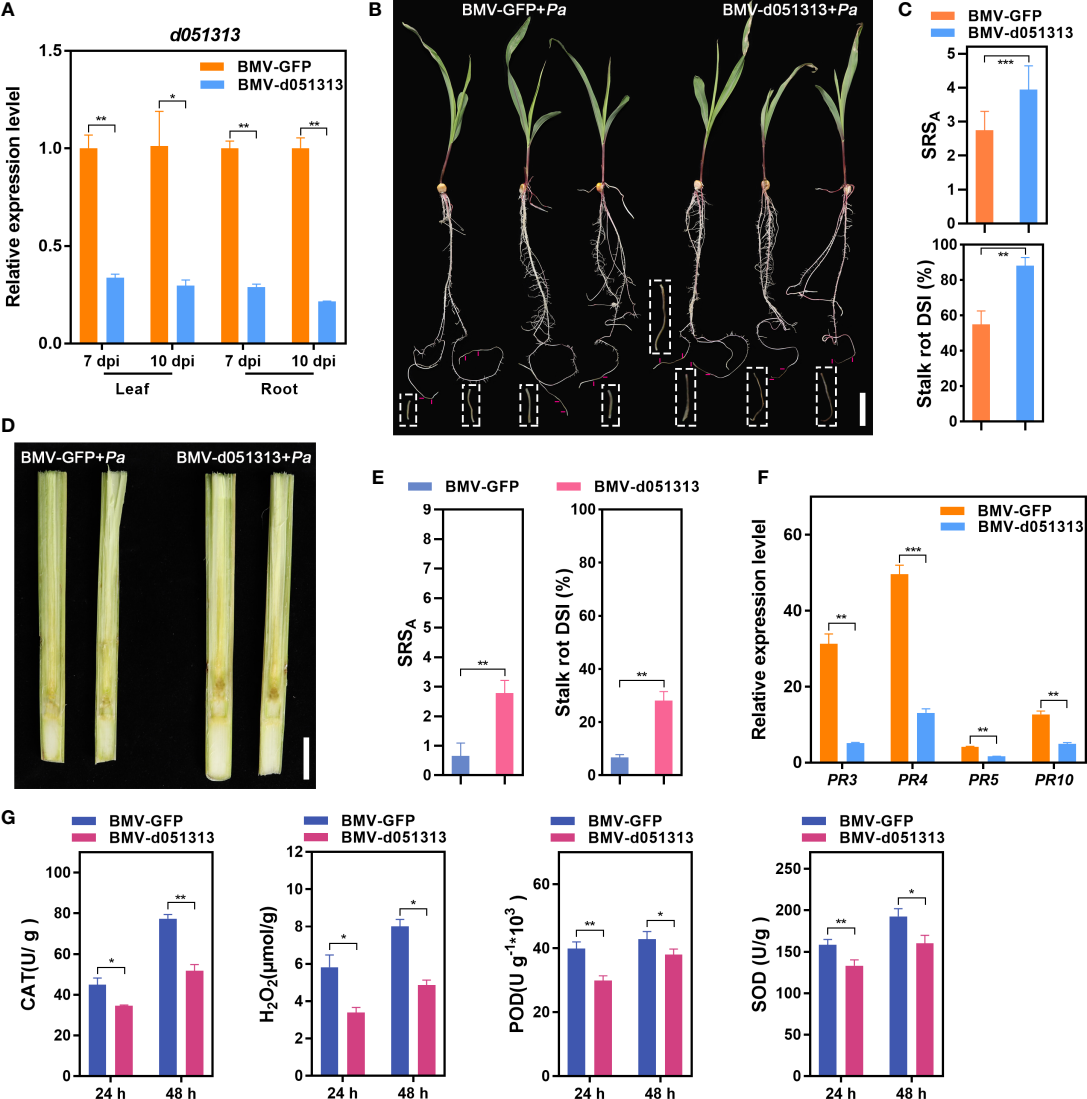
Transient silencing of LRR-RLK gene *Zm00001d051313* facilitates *Pythium aristosporum* infection in maize plants. **(A)** Silencing efficiency *Zm00001d051313* in the BMV-d051313 inoculated seedlings was evaluated by measuring the transcript levels of *Zm00001d051313* at 7 days post inoculation (dpi) with BMV-d051313, and 10 dpi, respectively. **(B)** At 7 dpi, the BMV-GFP and BMV-d051313 pre-inoculated cv. Va35 seedlings were challenged with *P. aristosporum*, and the inoculated seedling roots were recorded for symptoms in the diseased region at 48 h post inoculation (hpi). **(C)** The measurement of stalk rot score on average (SRS_A_) and stalk rot disease severity index (DSI) of *P. aristosporum*-inoculated maize seedling roots. **(D)** At 10 dpi, the stalks of BMV-GFP and BMV-d051313 pre-inoculated cv. Va35 plants used for parallel test were challenged with *P. aristosporum*, and the symptoms inside the stalks were recorded in the split internodes at 72 hpi. **(E)** The measurement of stalk rot score on average (SRS_A_) and stalk rot disease severity index (DSI) for *P. aristosporum*-inoculated maize stalks. **(F)** The expression profiles of pathogenesis-related (*PR*) genes, *ZmPR3*, *ZmPR4*, *ZmPR5*, and *ZmPR10* in the *Zm00001d051313*-silenced plants. **(G)** Quantification of CAT (catalase), H_2_O_2_, POD (peroxidase), and SOD (superoxide dismutase) levels in maize Va35 leaves of BMV-GFP controls and *Zm00001d051313* silencing plants at 24 h and 48 h after inoculation (hai) with *P. aristosporum*. Scale bars = 4 cm **(B)** and 2 cm **(D)**. Values are shown as the means ± SD. *P< 0.05, **P< 0.01, ***P< 0.001 (according to a paired Student’s *t*-test).

Among the differentially expressed candidate genes identified by GWAS, *Zm00001d051313* (d051313) encodes a potential LRR-RLK protein, which is a major class of PRRs modulating plant defense mechanism positively or negatively depending on the interaction (DeFalco and Zipfel, 2021). To test the potential roles of *Zm00001d051313* in regulating maize defense response against fungal infection, we examined the development of typical maize stalk rot symptoms in the roots and stalks of *Zm00001d051313* transient-silenced plants. We used an improved BMV-based gene silencing vector (Ding et al., 2018) to knock down *Zm00001d051313* expression through VIGS. Here, we introduced the VIGS vector BMV-d051313, harboring a *Zm00001d051313* gene-specific insert and control plasmid BMV-GFP into *Nicotina benthamiana* leaves for BMV virion proliferation. Then, we systemically infected the maize cv. Va35 seedlings with the partially purified BMV virion to transiently silence *Zm00001d051313* as previously described (Cao et al., 2012; Zhu et al., 2014). The qRT-PCR data demonstrated successful silencing of *Zm00001d051313*, with its expression in BMV-d051313 inoculated plants being ~21.5%–33% of that in the control plants (**Figure 5A**).

At 7 dpi, we challenged the root tips of BMV-GFP- and BMV-d051313-pre-inoculated maize Va35 seedlings with *P. aristosporum*. The *Zm00001d051313*-silenced roots exhibited more severe symptoms compared to the control plants at 48 hpi (**Figure 5B**), as confirmed by the SRS_A_ and DSI. Silencing *Zm00001d051313* in Va35 seedlings through VIGS led to a ~1.45-fold change of SRS_A_ and a 32.30% increase in DSI compared to the controls (**Figure 5C**). A parallel test also evaluated the effect of *Zm00001d051313* on the *P. aristosporum* infection inside maize stalks. After artificially challenging the stalks of the *Zm00001d051313*-silenced plants with *P. aristosporum* at 10 dpi, we split the internodes of the inoculated stalks to observe the stalk symptoms at 48 hpi. Similarly to the disease progression observed in the cv. Va35 seedling roots, transient silencing of *Zm00001d051313* also facilitated the infection of *P. aristosporum* inside the maize stalks (**Figures 5D, E**).

The enhanced susceptibility of *Zm00001d051313*-silenced maize seedlings to fungal infection prompted us to assess whether silencing affects the expression of maize defense-related genes. We analyzed the expression of pathogenesis-related (*PR*) genes like *ZmPR3*, *ZmPR4*, *ZmPR10*, and *ZmPR10* via qRT-PCR. The data showed a ~61%–84% reduction in the expression levels of *ZmPR3*, *ZmPR4*, *ZmPR5*, and *ZmPR10* in the *Zm00001d051313*-silenced seedlings compared to the BMV-GFP controls (**Figure 5F**). To determine the role of reactive oxygen species (ROS) in *Zm00001d051313*-mediated Pythium stalk rot resistance, we investigated H_2_O_2_ accumulation differences in *Zm00001d051313*-silenced and control maize seedlings post *P. aristosporum* infection. Generally, *Zm00001d051313*-silenced seedlings showed lower H_2_O_2_ levels upon *P. aristosporum* infection, while control seedlings showed higher activities of antioxidant enzymes such as CAT (catalase), POD (peroxidase), and SOD (superoxide dismutase) (**Figure 5G**).

## Discussion

### Identification of maize stalk rot resistance

Identification of disease resistance phenotypes is essential for selecting resistant maize varieties. The current methods for identifying maize stalk rot resistance can be divided into two main types: natural disease identification in the field and artificial inoculation identification. Compared to manual inoculation identification, natural pathogen identification methods in the field are associated with uncertainties, including the inability to guarantee consistent pathogen infection across batches of material, the possible existence of different species of pathogens in different environments, and the resulting influence of other potential pathogens on the phenotype (Li et al., 2001; Jiang et al., 2020). To solve these problems, we established and improved the stalk base drilling inoculation method for accurate identification of maize germplasm resistance to stalk rot (Duan et al., 2022). This method involves injecting a quantitative inoculum directly into the basal stalk of maize, ensuring consistent and adequate pathogen inoculation for each plant material. During the survey, the stalk of each plant is dissected to determine the area of disease incidence and grade according to the spread of necrotic lesion. This approach improves the resistance evaluation accuracy, prevents contamination from other potential organisms in the soil, and yields reliable results for resistance identification and evaluation of germplasm resources. Importantly, this method effectively reduces the false-positive SNPs due to different environments. Pythium stalk rot infection thrives in extended periods of hot (>32°C), wet, and humid weather, with the disease occurrence being particularly worse in fields with poor soil (Freije and Wise, 2016). In late July and early August 2021, Xinxiang experienced heavy rainfall-induced flooding, the highest ever figure recorded since 1951. We speculated that the flooding and accompanying high temperatures at the Xinxiang experimental station might favor the aggressiveness of *P. aristosporum*, leading to higher incidences of PASR in maize accessions planted in Xinxiang compared to those planted in Dancheng, despite using the same artificial inoculation protocols and grading standard in both experimental fields.

### Identification of SNPs significantly associated with stalk rot by GWAS

In maize disease resistance studies, GWAS has been successfully applied to identify QTL or genomic regions associated with resistance to vital maize diseases, including Fusarium ear rot (Yao et al., 2020), common rust (Olukolu et al., 2016), gray leaf spot (Kuki et al., 2018), northern corn leaf blight (Ding et al., 2015), southern corn leaf blight (Kump et al., 2011), rough dwarf (Chen et al., 2015), maize dwarf mosaic disease caused by sugarcane mosaic virus (Tao et al., 2013), and Fusarium stalk rot (Liu et al., 2021; Rashid et al., 2022). However, literature on application of GWAS in Pythium stalk rot disease resistance identification is currently unavailable.

We conducted a GWAS with 295 inbred maize lines to identify and validate genomic regions associated with PASR resistance. The panel was screened for PASR resistance in two environments using artificial inoculation methodology. To minimize false associations, we used six models (Blink, FarmCPU, GLM, MLM, MLMM, and SUPER) to identify significant SNPs associated with stalk rot resistance. We identified four SNPs (S4_ 153228905, S4_ 153228676, S4_ 153270388, and S4_ 153270407) associated with maize resistance to *P. aristosporum* in bin 4.06, consistent with the location range of stalk rot resistance genes *Rpi1* and *RpiX178-2* against *P. inflatum* (Yang et al., 2005; Duan et al., 2019). A previous study indicated that a key genomic region at 168 Mb on chromosome 6 was associated with Fusarium stalk rot resistance using GWAS and haplotype regression (Rashid et al., 2022). In this study, we identified a significantly associated SNP (S6_121809944) for PASR resistance in five models on chromosome 6 at 121 Mb. These crucial genomic regions warrant further investigation as potential candidate genes for controlling broad-spectrum resistance for stalk rot, pending future validation.

### Analysis of candidate gene *Zm00001d051313* associated with plant disease resistance

In plants, plasma membrane-associated pattern recognition receptors (PRRs) are deployed to sense conserved molecules derived from microbes, called pattern-associated molecular patterns (PAMPs), and molecules released by plant cells upon pathogen invasion (Chisholm et al., 2006). The recognition initiates a defense response known as pattern-triggered immunity (PTI) (Bigeard et al., 2015), which encompasses various cellular events like membrane ion flux changes, ROS and nitric oxide (NO) bursts, deposition of defense-related metabolites, modification of phytohormone concentrations, localized cell death, transcriptional changes, and others (Dodds and Rathjen, 2010). Most PRRs are receptor kinases (RKs) (Zipfel, 2014). LRR-RKs, a large family of several hundred proteins in most plant species, function as PRRs localized in the cell membrane, with the LRR domain in the extracellular space and the kinase domain located intracellularly (Bigeard et al., 2015). Maize plants possess LRR-RK genes in 16 clades (Man et al., 2020). Recently, an LRR-RK gene was linked to quantitative susceptibility to maize southern leaf blight (Chen et al., 2023); however, for the rest of the maize LRR-RK genes, their involvement in disease resistance is elusive. In this study, the functional annotation of *Zm00001d051313* revealed it as an encoded LRR family protein and localized to the cell membrane with a signal peptide structure (**Supplementary Figure S4**). Additionally, we found that silencing the expression of *Zm00001d051313* effectively reduced maize resistance to stalk rot (**Figures 5B, C**). Our recent findings indicated that *Zm00001d051313* was differentially expressed post infection with *F. verticillioides*, one of the most aggressive fungal pathogens causing maize stalk rot (Zhang et al., 2023). Jasmonic acid plays an important role in regulating biotic and abiotic stress responses, including plant responses to herbivorous arthropods, pathogenic bacteria, UV light, and ozone. *ZmJAZ2* (*Zm00001d027901*), one of the important regulators of jasmonate signaling, was induced significantly in response to *Puccinia polysora* Underw (Sun et al., 2021; Wang et al., 2022). Analysis of ChIP-seq data revealed the binding of *ZmJAZ2* (ZIM16) to the promoter region of *Zm00001d051313* (Tu et al., 2020), suggesting that *Zm00001d051313* may regulate maize’s antifungal defense response through the JAZ2-mediated jasmonate pathway.

In summary, we successfully identified the gene *Zm00001d051313* that is located on chromosome 4 and significantly associated with stalk rot resistance by GWAS analysis. We used VIGS to knock down the expression of *Zm00001d051313* in maize, verifying its function as a stalk rot tolerance gene. Our results demonstrated that *Zm00001d051313* is an LRR-RK gene that positively regulates stalk rot resistance in maize plants. Although it has important reference value for breeding resistant varieties in the future, the molecular mechanism by which this happens remains obscure; thus, the identification of the direct interaction of *Zm00001d051313* with its dimerization partners will help elucidate the underlying mechanism of its function.

## Data availability statement

The datasets presented in this study can be found in online repositories. The names of the repository/repositories and accession number(s) can be found in the article/**Supplementary Material**.

## Author contributions

YC, CD, and HL conceived and designed the study. MH, JY, SH, and TJ performed the experiments. MH, XZ, and SZ performed bioinformatics analysis. YC, MH, and XZ analyzed the data. LW, JL, LZ, HW, and XW participated in some experiments and provided help for the phenotypic measurement. YC, MH, and XZ wrote the manuscript. YC, CD, and HL revised the manuscript. All authors contributed to the article and approved the submitted version.
