# Supplementary material for: Genome-wide association study of maize resistance to Pythium aristosporum stalk rot
Source: Front Plant Sci. 2023 Aug 17;14:1239635. doi: 10.3389/fpls.2023.1239635 (PMC10470045; doi:10.3389/fpls.2023.1239635)
Supplement: Supplementary file 1 [file DataSheet_1.pdf]

Genome-wide association study of maize resistance to *Pythium aristosporum* stalk rot

Mengwei Hou[1,†], Yanyong Cao[1,†*], Xingrui Zhang[2,†], Shulin Zhang[3], Tengjiao Jia[1], Jiwei Yang[1], Shengbo Han[1], Lifeng Wang[1], Jingjing Li[1], Hao Wang[1], Lili Zhang[1], Xiaolin Wu[4], Canxing Duan[2, *], Huiyong Li[1,*]

[1]Institute of Cereal Crops, Henan Academy of Agricultural Sciences, Zhengzhou 450002, China.

[2]Institute of Crop Sciences, Chinese Academy of Agricultural Sciences, Beijing 100081, China.

[3] College of Biology and Food Engineering, Anyang Institute of Technology, Anyang 455099, China

[4] College of Life Science, Henan Agricultural University, Zhengzhou 450002, China

[†]These authors contributed equally to this work and share first authorship.

*Correspondence: Yanyong Cao (yanyongcao@126.com), Canxing Duan (duancanxing@caas.cn), Huiyong Li (lihuiyong1977@126.com)

SUPPLEMENTAL FIGURES

A

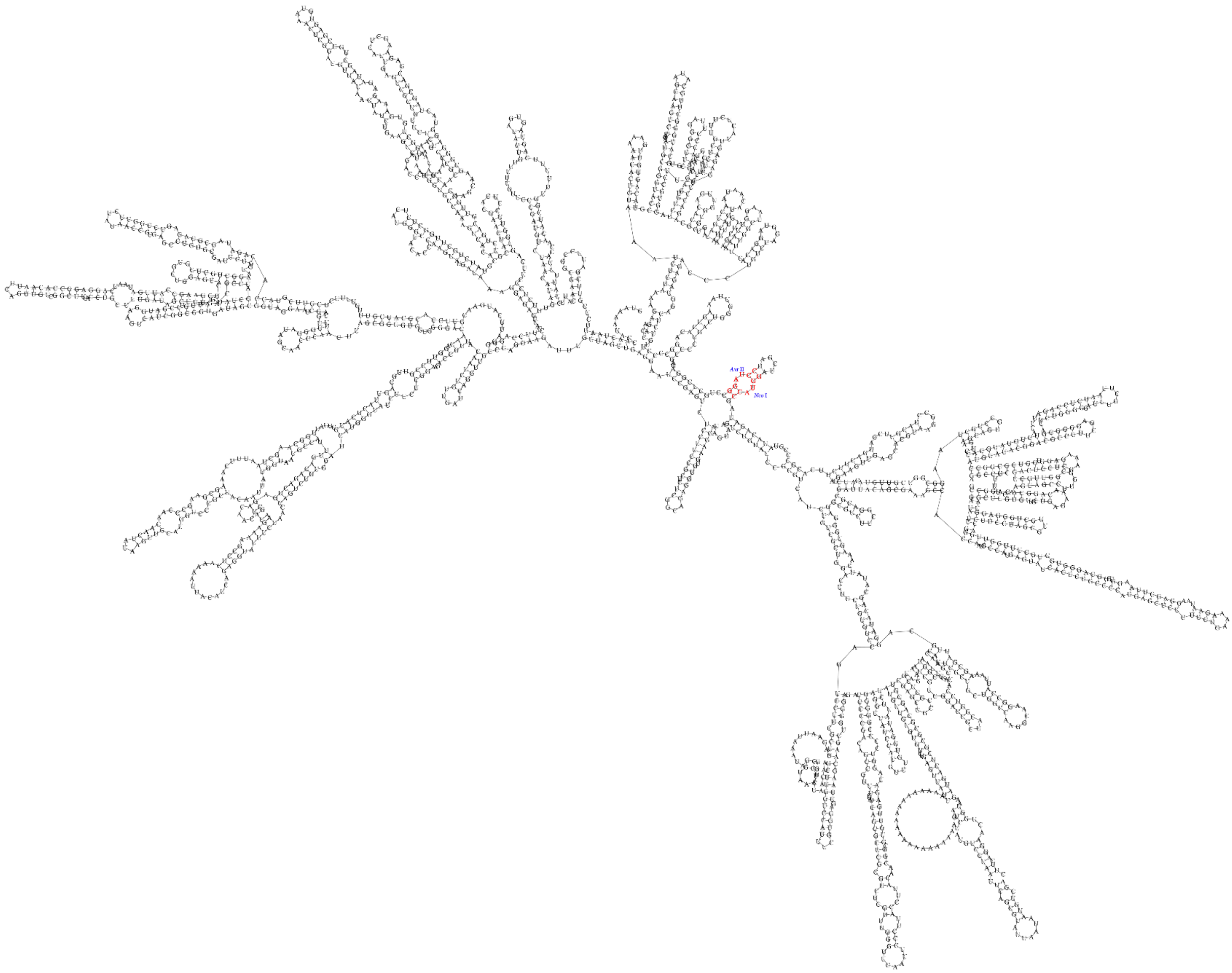

B

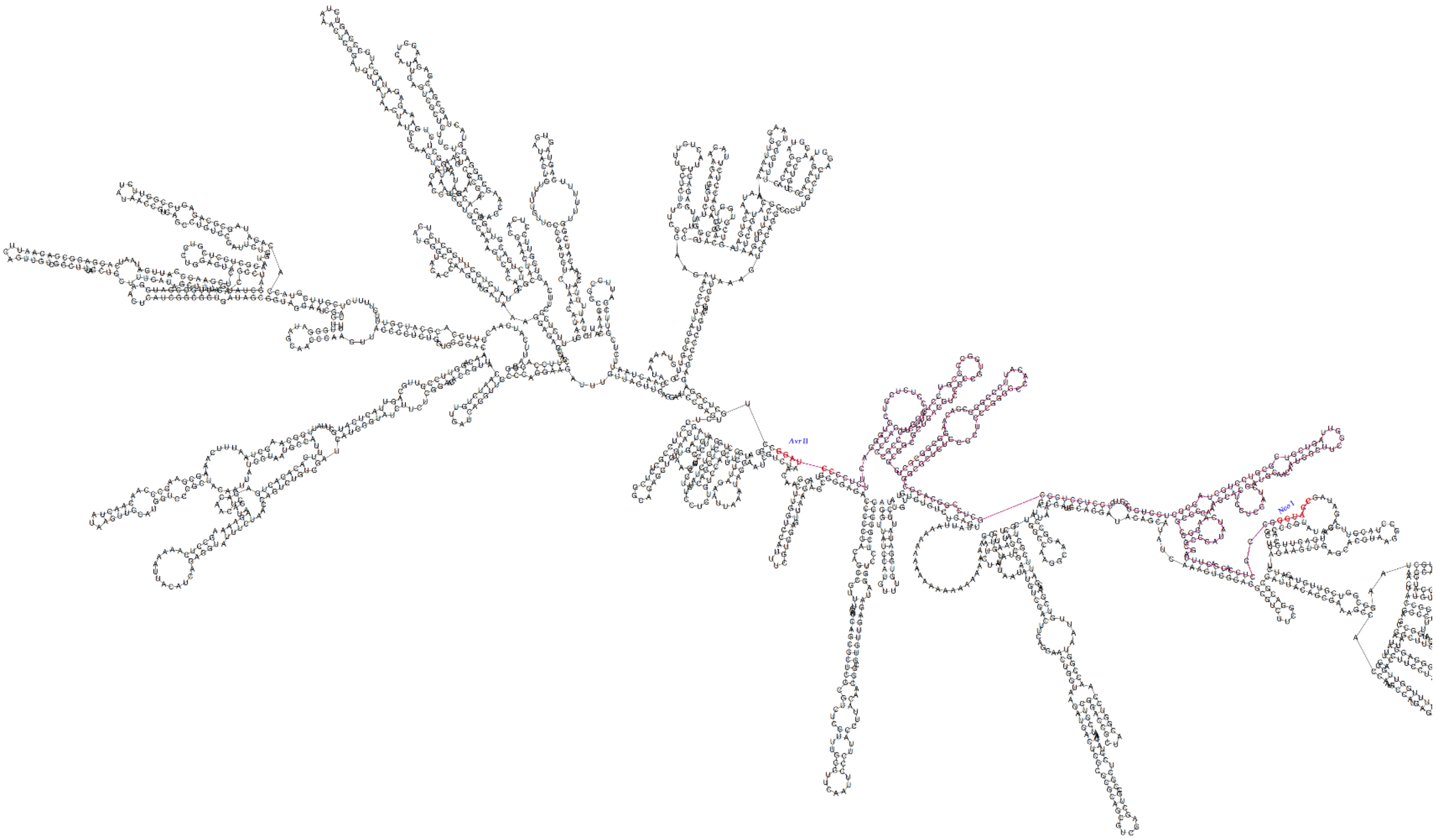

FIGURE S1 | Deep learning predicted BMVCP5 (+) strand RNA3 second structure without and with *Zm00001d051313* gene-specific fragment inserts. Full length (+) stands of BMV RNA3, without **(A)** or with gene fragment inserts representing *Zm00001d051313* **(B)** were folded with the MXFold2 Web server (http://www.dna.bio.keio.ac.jp/mxfold2/). Locations of the two cloning sites are indicated. Gene-specific fragment inserts were shown with magenta dashed lines.

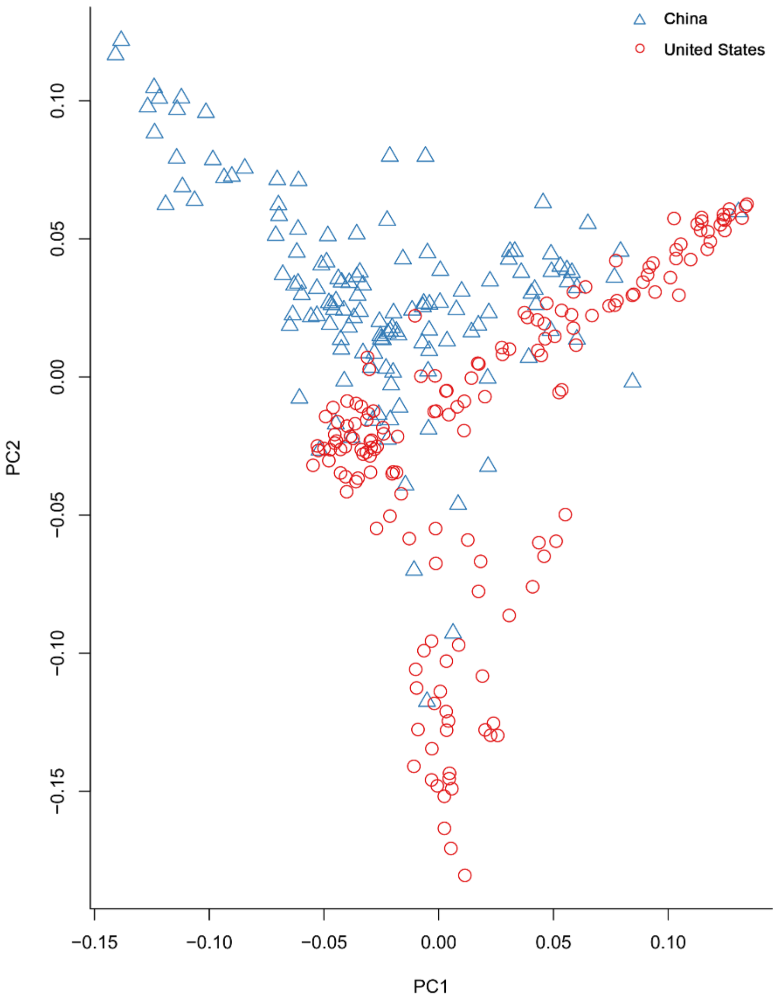

FIGURE S2 | Principal component (PC) analysis based on SNP genotypic data. Blue triangles and red circles indicate inbred lines that collected from China, and United States, respectively.

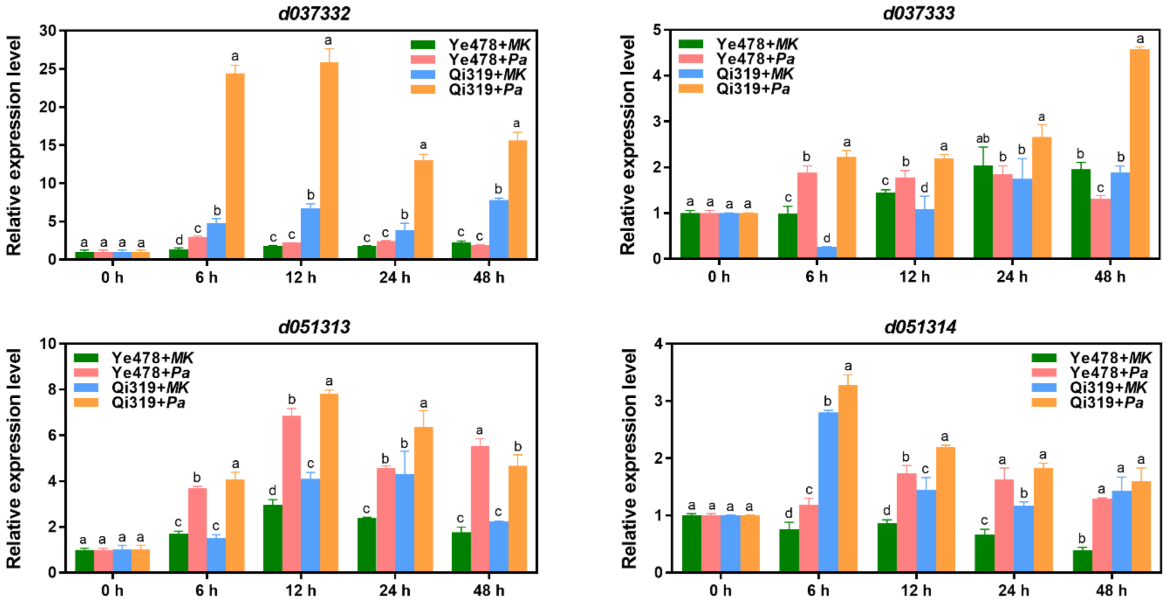

FIGURE S3 | Expression of the four candidate genes was quantified by qRT-PCR in two control inbred lines post *Pythium aristosporum* infection. Different letters on the columns show a significant difference ($P < 0.05$) as determined by the Tukey-Kramer test.

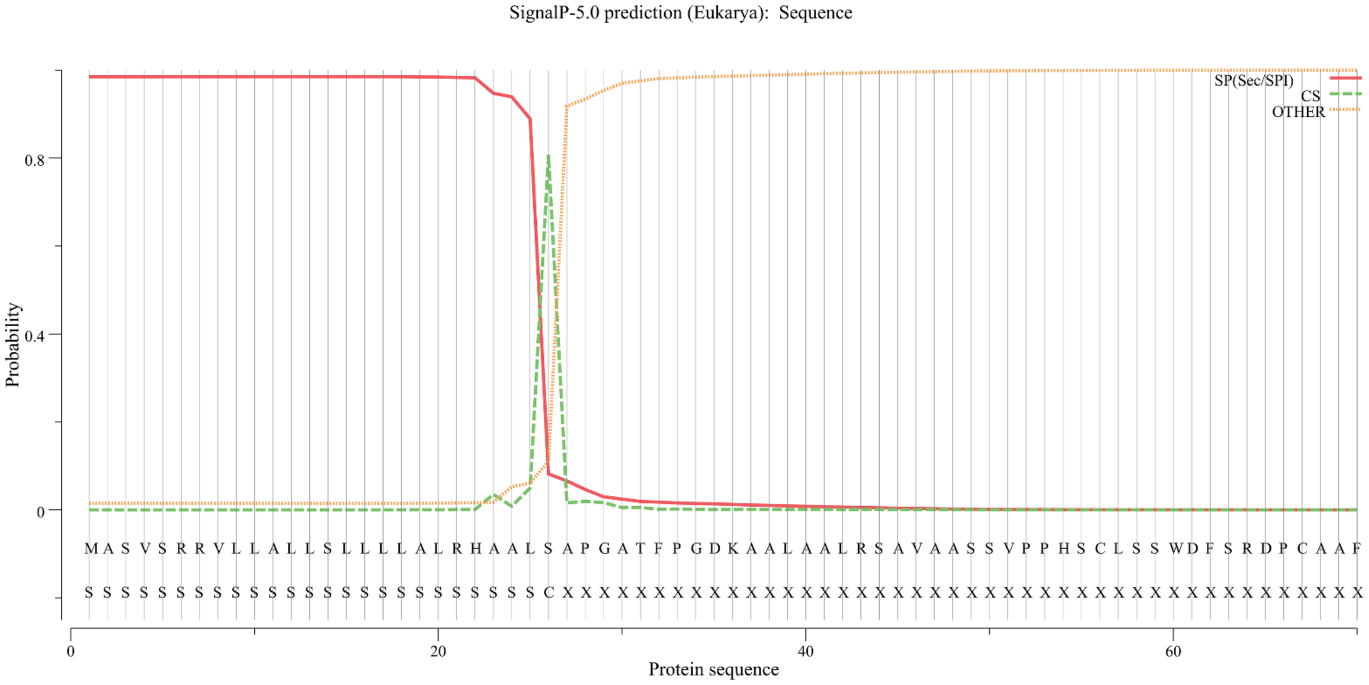

FIGURE S4 | Signal peptide prediction of proteins using SignalP-5.0 analysis. The first 70 residues from N-terminus of the each preprotein were selected for signal peptide prediction. Non-signal peptide (Other, yellow line) and Signal Peptide (SP, red line) moieties are reported as 0 (0%) to 1 (100%) probability. The cut site of SP (CS) is indicated as dashed green line.
